# Retraction Note: A Complete Formula of Ocean Surface Absolute Geostrophic Current

**DOI:** 10.1038/s41598-021-86517-3

**Published:** 2021-03-23

**Authors:** Peter C. Chu

**Affiliations:** Naval Ocean Analysis and Prediction Laboratory, Department of Oceanography, Naval Postgraduate School, Monterey, USA

Retraction of: *Scientific Reports* 10.1038/s41598-020-58458-w, published online 29 January 2020

The Author has retracted this Article.

In this study the Author treated the sea surface (S) and the marine geoid (N) as the two material surfaces and used the thermal wind relation between S and N to derive the formula for the ocean surface absolute geostrophic current with the intention to revise the oceanographic community's standard formula for surface geostrophic currents by allowing for nonzero flow at the level of the geoid. This approach was incorrect since the traditional formula currently used by the oceanographic community makes no such assumption, and is not derived by integrating from the geoid to the sea surface. Instead, it is derived via a direct relationship between sea surface elevation changes relative to N.

The commonly-used description of the geoid as “the sea surface when there is no flow” refers to a hypothetical situation. If the sea surface elevation (S) was governed only by geostrophy and coincided with one particular equipotential taken as the geoid, then there would be no flow on this surface. The geoid is an imaginary surface that could be either above or below the actual sea surface. An additional error was present in that the derived formula had an opposite sign in dynamic ocean topography (D) term.

The Author agrees with the retraction and the wording of this retraction notice.

